# Fostering guardians for frontline medical disputes: a government-led medical dispute mediator training program in Taiwan

**DOI:** 10.1186/s12913-022-08909-z

**Published:** 2022-12-05

**Authors:** Wan-Ting Chen, Yu-Ying Huang, Wen-Wen Chen, Yueh-Ping Liu, Chung-Liang Shih, Yi-Chih Shiao, Chih-Chia Wang

**Affiliations:** 1 Department of Psychiatry, Tri-Service General Hospital and School of Medicine, National Defense Medical Center, No.325, Sec. 2, Chenggong Rd., Neihu Dist., Taipei City, 114 Taiwan; 2 Taiwan Drug Relief Foundation, 10F., No. 22, Aiguo E. Rd., Zhongzheng Dist., Taipei City, 100 Taiwan; 3 Department of Medical Affairs, Ministry of Health and Welfare, No. 488, Section 6, Zhongxiao E Rd, Nangang District, Taipei City, 115 Taiwan; 4 Ministry of Health and Welfare, No. 488, Section 6, Zhongxiao E Rd, Nangang District, Taipei City, 115 Taiwan; 5 Department of Family and Community Medicine, Tri-Service General Hospital and School of Medicine, National Defense Medical Center, No.325, Sec. 2, Chenggong Rd., Neihu Dist., Taipei City, 114 Taiwan; 6 College of Law, National Chengchi University, No.64, Sec.2, ZhiNan Rd., Wenshan District, Taipei City, 116 Taiwan

**Keywords:** Medical dispute, Interdisciplinary, Mediator training, Government-led

## Abstract

**Background:**

Mediation is increasingly used for medical dispute resolution, and the particularity of such mediation necessitates specialized training. In response to the promotion of compulsory mediation ahead of a legislation in Taiwan, we invited experts with an interdisciplinary team to design a case-based mediator training workshop. Our study aimed to investigate the learning outcomes of trainees and analyze their perspectives.

**Methods:**

We recruited 129 trainees of a non-probability convenience sample who served as mediators or have dealt with medical dispute-related cases to undergo 2.5 h of lectures (introduction; procedure; roles of two mediators; principles and techniques of mediation; dispute arrangement; and issue analysis) and 1.5 h of case-based exercises. An after-class survey was conducted using a 4-point Likert-type scale to evaluate trainees’ viewpoints and learning outcomes. A total of 104 questionnaires were collected (response rate: 80.6%).

**Results:**

The professions of the participants were medical (56%), law (16%), and administration and others (28%). Males considered the course more helpful (3.79 vs. 3.63, *p* = 0.053) and more important (3.88 vs. 3.74, *p* = 0.042) than did females. Participants with a legal background scored the highest in helpfulness (3.84), followed by medical (3.74) and administrative (3.63) professionals. Medical and administrative professionals scored the highest (3.85) and lowest (3.76), respectively, on importance. Respondents with more than 10 years (3.81) and less than 1 year (3.79) of experience produced higher scores in helpfulness. Respondents with 1–5 years of experience (3.68) were found to be less likely to agree with the practical importance of course content compared with other groups of trainees. Administrative professionals obtained the highest scores (89.68) in written examinations.

**Conclusions:**

There are variations in mediators’ perspectives based on gender, occupation, and work experience. Our nationwide mediation training workshop can be utilized to cultivate capabilities of mediators for handling medical disputes to achieve the goal of non-litigation in medical disputes.

**Supplementary Information:**

The online version contains supplementary material available at 10.1186/s12913-022-08909-z.

## Background

With the increase in medico-legal controversies in recent decades [1, 2], a growing number of clinicians are compelled to struggle in medical disputes, conflicts, or even litigations [3]. Although litigation is expected to bring justice to both clinicians and patients, it is inefficient in settling medical disputes comprehensively [4]. Furthermore, it may even damage doctor–patient relationships [5, 6]. To achieve conflict resolution without litigation, alternative dispute resolution (ADR) has been applied for cases of medical malpractice [7]. ADR, with benefits like efficiency in terms of time and cost [8], leads to the improved satisfaction of plaintiffs and defendants [9]. Among ADR methods, mediation is a prominent tool for reaching amicable settlements. In this process, a neutral party assists two parties in communicating and negotiating while ensuring the observance of the rights and autonomy of the two parties [7].

To incorporate ADR into the legislative system, the Taiwanese government has bolstered the Prevention and Resolution of Medical Malpractice (PRM) Act since 2000, thus achieving the following goals: mediating litigation that protects the rights and interests of patients; promoting harmony between doctors and patients; and improving the quality of medical care. Since then, in Taiwan, patients would be compulsorily referred to mediation before entering judicial proceedings (see Supplementary 1). For handling medical disputes successfully, cross-disciplinary cooperation between medical and legal experts is necessary [10]. Therefore, since 2012, a dual-mediator model (DMM)—that is, one mediator from the medical profession and the other from the legal profession—has been applied in district courts [11], with the aim of simultaneously clarifying medical malpractice facts and legal issues [12]. This model’s application has led to many successful resolutions of malpractice claims in Taiwan [11].

Considering that the PRM Act clearly states that mediation is compulsorily prior to legal proceedings in medical disputes, such a change must be communicated to all mediation staff and related practitioners. A workshop was deemed necessary to efficiently execute this process [13], after which the Taiwan Drug Relief Foundation (TDRF), with the aid of the Ministry of Health and Welfare (MOHW), was designated to organize such a workshop. The workshop’s purpose was to ensure that all frontline mediators have a comprehensive understanding of this new policy’s direction, enabling them to deal with medical disputes more effectively under the renewed healthcare system. Therefore, the present study examines the effectiveness of a government-led mediation training workshop, with specific focus on the participants’ degree of satisfaction with the learning experience and their preferences with different backgrounds.

## Methods

### Rapid review

Rapid review is an efficient approach that supports health policymaking in establishing relevant regulations by providing systemic evidence [14]. With the increasing prevalence of non-litigation, several countries have established mediation systems to handle medical disputes. For example, the German government has established specialized committees of appraisal (Gutachterkommissionen) and mediation (Schlichtungsstellen) as an alternative to litigation [15], while the South Korean government has formulated the Korea Medical Dispute Mediation and Arbitration Board to quickly and smoothly resolve such conflicts [16]. In Taiwan, this process is conducted on a case-by-case basis, with the aid of mediators. Our course is based on the teaching plans of foreign training institutions such as the Harvard Negotiation and Mediation Clinical Program in the United States and the Singapore Mediation Centre. In response to the new legislation, the course content includes the DMM.

### Lecture design

A meeting of experts was conducted to formulate the training curriculum. The curriculum design was based on both former governmental and non-governmental guidelines for medical dispute mediation, including court-based mediation committee training courses and arbitration association mediation courses. All 14 experts in this program were formally invited through the official recruitment process of the TDRF based on purposive sampling. The experts comprised specialists in different fields, some of whom were involved in the legislative process of the PRM Act, including physicians with both a medical degree (M.D.) and a Master of Laws (LL.M.) degree, judges who have overseen medical disputes, lawyers with extensive litigation experience in medical disputes, and scholars in medical law development and public health communities.

### Participants

Due to the scarcity of mediators, our research is based on convenience samples using in non-probability sampling manner. Recruitment of the convenience sample was primarily done via social media platforms (TDRF websites and Facebook) and official documents issued to institutes. Eligible participants who signed registration form were contacted afterwards via e-mail, including those who served as mediators for the coordination of medical disputes in county/city health bureaus and courts; health specialists worked in medical institutions; others worked in non-government organizations, providing legal counseling to patients/family members, etc. This study aims to gather information on professions with different working backgrounds that can represent the applicability of the course. As pilot studies have comparatively small sample sizes and have variables in sample size calculation, there was no predetermined sample size [17, 18].

### Procedures/Intervention

A four-hour medical dispute mediation training workshop was designed (see Supplementary 2), including [1] 2.5 h lectures on the background of the PRM Act, core meanings of prevention and resolution, and practical aspects of effective mediation and [2] a 1.5 h field exercise with a situation-based scenario to simulate the mediation process. Four workshops each were held in the northern, central, southern, and eastern districts of Taiwan from June to October 2019. The experts in the legislative process were responsible for formulating and teaching educational content. For reference, the official teaching materials were published in 2021 on the MOHW website [12].

### Ethics statement

The study was approved by the Institutional Review Board of the Tri-Service General Hospital, National Defense Medical Center (TSGHIRB No.: E202216022). This study was conducted in line with the Declaration of Helsinki, and informed consent was obtained before conducting research.

### Data collection and analysis

A questionnaire comprising 12 questions was created after the completion of training to evaluate and assess trainees’ perspectives on the degree of helpfulness of the course and the importance of the training content to practice. Items were rated using a 4-point Likert-type scale (1 = completely disagree, 2 = disagree, 3 = agree, 4 = completely agree). As for its validity, a consensus meeting was held by five professionals in mediation for medical disputes from different backgrounds, including two senior clinical physicians, two lawyers, and a judge, to formulate the questionnaire and ensure that all the items in the questionnaire cover all facets of the construct being measured. Additionally, we conducted post-class tests on the six major themes of the course. The respondents participated in the survey voluntarily. The survey did not collect participants’ personal data, thus ensuring their anonymity.

### Statistical analysis

Cronbach's α coefficient, a measure of internal consistency, was utilized to examine the reliability of the questionnaire. Value equal to or higher than 0.7 or was thought to be satisfactory [19]. Data analysis was performed using the version 18.0 of Statistical Product and Service Solutions for Windows (SPSS Inc., Chicago, Illinois, United States).

## Results

We conducted four mediation training courses across Taiwan (northern, middle, southern, and eastern regions) in 2019 with a total of 129 participants (north: 38, middle: 30, south: 33, and east: 33), and 104 valid questionnaires were effectively retrieved with effective recovery of 80.6%. The reliability of the questionnaire was assessed using the Cronbach’s alpha coefficient (0.94 for importance; 0.95 for helpfulness). Table 1 presents participants’ demographics and the classification of working places, their status as mediators (i.e., currently engaged or not), and years of experience.

**Table 1:** Characteristics of study participants

**Characteristics of Study Participants**	n(%) (*N* = 104)
**Region**	
North	28 (26.9)
Central	19 (18.3)
South	30 (28.8)
East	27 (26)
**Gender**	
Male	58 (55.8)
Female	45 (43.3)
**Profession**	
Medicine	58 (55.8)
Administration	21 (20.2)
Law	17 (16.3)
Others	8 (7.7)
**Service Units**	
Medical Center	15 (14.4)
Regional hospital	18 (17.3)
Local Community Hospitals	12 (11.5)
Clinics	15 (14.4)
Law Firm	3 (2.9)
Government Agencies	23 (22.1)
Consortium	5 (4.8)
Others	13 (12.5)
**Positions**	
Current Mediation Member	39 (37.5)
Mediation Reserve Talents	33 (31.7)
Administrative Public Affairs	30 (28.8)
Former Mediation (Office) Member	2 (1.9)
**Mediation Practice Experience**	
Less than 1 Year	51 (49)
1–5 Years	19 (18.3)
5–10 Years	11 (10.6)
More than 10 Years	23 (22.1)

In general, the respondents held a positive view of the mediation course. Approximately 95% of the respondents consider the course helpful or very helpful, and 90% reported the content to be important or very important to practice. A gender subgroup analysis illustrated that males considered the course to be more helpful than females (3.79 vs. 3.63, *p* = 0.053), especially in terms of the medical mediation process, the role of dual mediators, and related principles and skills. Similarly, more males assessed the course as important (3.88 vs. 3.74, *p* = 0.042) than females, especially with regards to the introduction of the medical mediation mechanism, procedures, and the role of dual mediators (Table 2).

**Table 2:** Evaluation of the helpfulness and importance of different genders to the workshop

		**Helpfulness**			**Importance**	
	Male (*n* = 58)	Female (*n* = 45)	*p* value	Male (*n* = 58)	Female (*n* = 45)	*p* value
Introduction to medical dispute mediation mechanism	3.81 ± 0.40	3.67 ± 0.48	0.106	3.93 ± 0.26	3.73 ± 0.45	0.010*
Medical dispute mediation procedure	3.86 ± 0.35	3.67 ± 0.48	0.023*	3.93 ± 0.26	3.78 ± 0.42	0.035*
Role of the dual-mediators	3.81 ± 0.4	3.62 ± 0.49	0.039*	3.93 ± 0.26	3.73 ± 0.45	0.010*
Mediation principles and techniques	3.81 ± 0.40	3.62 ± 0.49	0.039*	3.88 ± 0.33	3.76 ± 0.43	0.116
Issues of law	3.74 ± 0.48	3.62 ± 0.49	0.218	3.84 ± 0.41	3.73 ± 0.45	0.197
Legal analysis	3.72 ± 0.49	3.6 ± 0.50	0.206	3.81 ± 0.48	3.71 ± 0.46	0.289
**Average**	**3.79 ± 0.37**	**3.63 ± 0.44**	**0.053**	**3.88 ± 0.26**	**3.74 ± 0.42**	0.042*

A subgroup analysis of professions demonstrated that participants from legal backgrounds scored the highest in helpfulness (3.84), followed by those from medical (3.74), administrative (3.63), and other (3.65) backgrounds. In terms of importance, the medical and administrative professions, respectively, scored the highest (3.85) and lowest (3.76) (Table 3). When classified according to experience, respondents with more than 10 years (3.81) and less than 1 year (3.79) of experience scored the highest in helpfulness. Participants with 1–5 and 5–10 years of experience rated the workshop as less helpful (*p* = 0.043). Trainees with 1–5 years of mediation experience (3.68) were less likely to agree with the practical importance of course content compared with other groups of trainees (Table 4). We also performed subgroup analysis of regions, service units, and positions; however, there is no significant difference among them.

**Table 3:** Evaluation of the helpfulness and importance of different professional backgrounds to the workshop

	**Helpfulness**					**Importance**				
	Medicine (*n* = 58)	Law (*n* = 17)	Administration (*n* = 21)	Others (*n* = 8)	*p* value	Medicine (*n* = 58)	Law (*n* = 17)	Administration (*n* = 21)	Others (*n* = 8)	*p* value
Introduction to medical dispute mediation mechanism	3.79 ± 0.41	3.76 ± 0.44	3.62 ± 0.50	3.75 ± 0.46	0.483	3.9 ± 0.31	3.82 ± 0.39	3.76 ± 0.44	3.75 ± 0.46	0.415
Medical dispute mediation procedure	3.81 ± 0.40	3.88 ± 0.33	3.67 ± 0.48	3.63 ± 0.52	0.265	3.88 ± 0.33	3.88 ± 0.33	3.81 ± 0.4	3.88 ± 0.35	0.877
Role of the dual-mediators	3.74 ± 0.44	3.88 ± 0.33	3.62 ± 0.50	3.63 ± 0.52	0.29	3.86 ± 0.35	3.88 ± 0.33	3.76 ± 0.44	3.88 ± 0.35	0.696
Mediation principles and techniques	3.72 ± 0.45	3.88 ± 0.33	3.67 ± 0.48	3.63 ± 0.52	0.418	3.84 ± 0.37	3.82 ± 0.39	3.76 ± 0.44	3.88 ± 0.35	0.836
Issues of law	3.69 ± 0.50	3.82 ± 0.39	3.57 ± 0.51	3.75 ± 0.46	0.45	3.83 ± 0.42	3.71 ± 0.47	3.76 ± 0.44	3.88 ± 0.35	0.692
Legal analysis	3.67 ± 0.51	3.82 ± 0.39	3.62 ± 0.50	3.5 ± 0.54	0.422	3.79 ± 0.45	3.76 ± 0.56	3.71 ± 0.46	3.75 ± 0.46	0.93
**Average**	**3.74 ± 0.40**	**3.84 ± 0.34**	**3.63 ± 0.45**	**3.65 ± 0.46**	**0.392**	**3.85 ± 0.32**	**3.81 ± 0.34**	**3.76 ± 0.41**	**3.83 ± 0.36**	**0.794**

**Table 4:** Evaluation of the helpfulness and importance of different mediation practice experiences to the workshop

	**Helpfulness**					**Importance**				
	Less than 1 year (*n* = 51)	1–5 years (*n* = 19)	5–10 years (*n* = 11)	More than 10 years (*n* = 23)	*p* value	Less than 1 year (*n* = 51)	1–5 years (*n* = 19)	5–10 years (*n* = 11)	More than 10 years (*n* = 23)	*p* value
Introduction to medical dispute mediation mechanism	3.82 ± 0.39	3.58 ± 0.51	3.45 ± 0.52	3.87 ± 0.34	0.009**	3.88 ± 0.33	3.74 ± 0.45	3.82 ± 0.4	3.87 ± 0.34	0.5
Medical dispute mediation procedure	3.84 ± 0.37	3.68 ± 0.48	3.55 ± 0.52	3.83 ± 0.39	0.114	3.88 ± 0.33	3.74 ± 0.45	4 ± 0	3.87 ± 0.34	0.214
Role of the dual-mediators	3.78 ± 0.42	3.63 ± 0.50	3.55 ± 0.52	3.78 ± 0.42	0.276	3.84 ± 0.37	3.68 ± 0.48	4 ± 0	3.91 ± 0.29	0.085
Mediation principles and techniques	3.8 ± 0.40	3.63 ± 0.50	3.45 ± 0.52	3.78 ± 0.42	0.075	3.86 ± 0.35	3.68 ± 0.48	3.91 ± 0.3	3.83 ± 0.39	0.305
Issues of law	3.75 ± 0.44	3.53 ± 0.61	3.45 ± 0.52	3.83 ± 0.39	0.062	3.86 ± 0.35	3.63 ± 0.6	3.82 ± 0.4	3.78 ± 0.42	0.251
Legal analysis	3.73 ± 0.45	3.53 ± 0.61	3.45 ± 0.52	3.78 ± 0.42	0.132	3.8 ± 0.4	3.63 ± 0.6	3.82 ± 0.4	3.78 ± 0.52	0.564
**Average**	**3.79 ± 0.36**	**3.60 ± 0.47**	**3.48 ± 0.49**	**3.81 ± 0.37**	0.043*	**3.86 ± 0.32**	**3.68 ± 0.48**	**3.89 ± 0.19**	**3.84 ± 0.32**	**0.254**

The average test score was approximately 85. Females obtained higher grades than did men (85.93 vs. 81.03, *p* = 0.22). Respondents with 1–5 years of experiences obtained higher scores. However, no significant difference was observed in the length of experience. On classifying by profession, we found that the administrative professions obtained the highest scores (89.68), followed by the legal (84.31), medical (83.91), and other (60.42) professions (*p* = 0.004; Table 5).

**Table 5:** Number of participants who answered correctly in post test

	Gender		Mediation Practice Experience				Profession			
	Male	Female	less than 1 year	1 to 5 years	5 to 10 years	More than 10 years	Medicine	Law	Administration	Others
Medical dispute mediation law	33	37	38	16	7	10	42	11	18	0
Medical dispute mediation mechanism	44	38	42	17	5	19	45	14	19	5
Principles of mediation	47	34	37	17	10	18	45	14	17	6
Mediation procedures	52	38	46	15	10	20	51	16	19	5
Roles of mediators	53	43	47	18	10	22	55	16	20	6
Reporting the process of medication	53	42	49	16	11	20	54	15	20	7
**Average**	**81.03**	**85.93**	**84.64**	**86.84**	**80.30**	**78.99**	**83.91**	**84.31**	**89.68**	**60.42**
*p* **value**	**0.22**		**0.549**				0.004**			

## Discussion

The government-led medical dispute mediation training workshop in this study aims to systemically enhance mediators’ knowledge regarding the new mediation stages of the PRM Act and polish professional knowledge and skills. In summary, the trainees acknowledged that the workshop was helpful and that the content was important for practice. However, discrepancies were noted in the degree of agreement among trainees with different gender, occupations, and work experiences. Our study found that participants who were male, from legal professions, and mediators with less than 1 year of experience and more than 10 years of experience provided high ratings for helpfulness. Regarding the importance of the content, more participants who were male and from medical professions considered the course important. In a post-class quiz, an administrative professional obtained the highest scores. There were discrepancies in the degree of helpfulness and importance of the course based on gender. It has long been debated whether gender is a variable of mediation style or effectiveness. Some studies have suggested that gender can cause differences in mediation style and content [20], including in terms of communication techniques and the formulation types used in the mediation process [21]. Thus, a differentiated instruction of the mediation course can be provided to adjust for the needs of mediators from contrasting backgrounds.

The variations in the degrees of importance and helpfulness among different groups may have originate from the variances in working environments and distinctive roles. One previous study suggested that lawyers and mental health practitioners should use multiform approaches based on different orientations to practice and present assumptions [22]. Further, it has been argued that variations in mediation models reflect the styles and concerns of various professional backgrounds [23]. Differences in job contents give rise to differences in familiarity with specific topics, which may constitute a possible explanation for diversity in test scores. Executive trainees obtaining the highest test scores reflect not only their thorough understanding of the course but also their familiarity with laws and regulations on related topics. As successful mediation requires the cooperation of people from multiple disciplines, participants from different professional backgrounds working together can promote cross-disciplinary learning [24]. It not only provides practical information for legal mediation committees in understanding the complexity, high risk, and unpredictability of medical disputes [25] but also enhances medical staff’s capacity for addressing conflicts in terms of legality. Participants can stimulate cognition from multiple perspectives through knowledge-sharing and collaboration across disciplines by working with each other [26, 27].

The participation rate of medical staff was significantly higher than that of the legal staff. Coincidentally, medical professions scored higher in the attribution of the level of importance of the study. The high participation rate and scores for the importance of medical dispute mediation may partially be due to the concerns of health professionals about medical disputes. Moreover, the difference in the participation rates may also reflect the distinct viewpoints between legal and medical professions regarding attending mediation training courses. The learning motivation of the medical professionals lies in the anxiety of being indicted, such that physicians feel uncomfortable with deficient knowledge on medical malpractice [28]. Indeed, although physicians always prioritize patients’ interest in medical decision making, they may generate conflict with patients because of divergence in values [29, 30]. The entrenched expectation gap between physicians and patients may lead to misunderstandings in patients when they encounter unexpected medical results, resulting in medical disputes [31].Thus, medical personnel consider strengthening their abilities to resolve medical disputes owing to the surge of cases of medical disputes [1]. Nevertheless, legal professionals’ learning motivation may also originate from an intrinsic curiosity in comprehending a specific type of litigation.

Our findings suggest the exigent need to enhance medical staff’s education in medical law. The high participation rate of medical professionals reflects a general lack of direct experience in dispute resolution or mediation training. Conflict management not only enhances the ability of the medical staff to address disputes but also enhances information communication and management among medical teams [32]. Thus, an understanding of the mediation process may help medical staff to alleviate the psychological pressure of encountering such disputes. Moreover, it enables a smooth collaboration with lawyers during the legal process [33]. Cooperation between physicians and legal professionals is required not only for litigation but also for the improvement of medical care. For instance, a medical–legal partnership in Atlanta trains future physicians to coordinate with lawyers to help improve the health status and legal rights of patients [34]. As such, medical education should steer toward systematically developing programs for medical law education [35], while pertinent training courses can be integrated into undergraduate, postgraduate, and continuing education. For example, medico-legal education, such as court-based learning, visiting actual courts, and creating opportunities to communicate with legal professionals, can help students enrich their knowledge in medical ethics and law and remove negative stereotypes about medical disputes [36].

The current course is a nation-drive key project to promote the non-litigation of medical disputes in line with the PRM Act. The legislative purpose of this act is to resolve confrontations between doctors and patients arising from medical disputes, ensure the quality and quantity of health professionals, and reduce the probability of defensive medicine. This mediation course is part of the MOHW project for promoting non-litigation education and encouraging mediators to put it into practice. These trained mediators are eventually included in the government’s mediation talent database. Such an approach enables the government to offer continuum education to mediators. Continuum education not only correlates with high settlement rates [37] but also provides mediators with updated knowledge of laws and regulations and expands cooperative resources.

This study has its limitations. First, our sample is a self-selected convenience sample and not representative of the general population. This potential deficiency of representativeness limits the probability of generalizability. Second, we lacked the pre-test data to evaluate the trainees’ attitude and knowledge performance because it was a preliminary study of the lecture design. Another drawback is that although the results indicate that the mediators recognized the helpfulness and importance of our course, less could be inferred about their behavioral changes. Therefore, longitudinal research with a larger population size is required to determine the long-term development of trainees. Currently, a multi-stage, customized workshop for mediation talent is under planning. We are planning to incorporate various teaching methods into the courses to enhance the quality of learning contents, such as the application of problem-based learning to cultivate trainees’ ability in searching for and integrating information [38]. Assessment tools, including professional activities for faculty to make competency-based decisions on the level of supervision [39] or milestones [40], can also be utilized to assess whether trainees can perform independently during apprenticeship.

## Conclusions

The government-led medical mediation workshop in this study systemically promoted the legislation for non-litigation to medicators, integrating mediators’ experience into the course while transforming their mediation skills. Variations were found in terms of gender, occupations, and work experiences regarding preferences for mediation courses’ design. Of which, medical personnel had high participance rate in the medical dispute mediation course and considered the course important. A customized training course should be established to improve the effectiveness accordingly.

## Supplementary Information

**Additional file 1.**

## Data Availability

The datasets used and/or analyzed during the current study are available from the corresponding author on reasonable request.

## Abbreviations

MOHW: Ministry of Health and Welfare
TDRF: Taiwan Drug Relief Foundation
PRM: Prevention and resolution of medical malpractice
DMM: Dual-mediator model
