# Supplementary material for: Fostering guardians for frontline medical disputes: a government-led medical dispute mediator training program in Taiwan
Source: BMC Health Serv Res. 2022 Dec 5;22:1478. doi: 10.1186/s12913-022-08909-z (PMC9720959; doi:10.1186/s12913-022-08909-z)
Supplement: Supplementary file 1 — Additional file 1. [file 12913_2022_8909_MOESM1_ESM.docx]

Supplementary Appendix

This appendix has been provided by the authors to give readers additional information about the work.

**Supplementary materials**

**Fostering Guardians for Frontline Medical Disputes: A Government-Led Medical Dispute Mediator Training Program in Taiwan**

Wan-Ting Chen^a^, Yu-Ying Huang^b^, Wen-Wen Chen^c^, Yueh-Ping Liu^d^, Chung-Liang Shih^e^, Yi-Chih Shiao^f^, and Chih-Chia Wang^g^*

*^a^Department of Psychiatry, Tri-Service General Hospital and School of Medicine, National Defense Medical Center, No.325, Sec. 2, Chenggong Rd., Neihu Dist., Taipei City, 114, Taiwan, friend1584@gmail.com; ^b^Taiwan Drug Relief Foundation, 10F., No. 22, Aiguo E. Rd., Zhongzheng Dist., Taipei City, 100, Taiwan, Managing Lawyer of Huang’s Law Office, yuyinghuang1007@gmail.com; ^c^**Taiwan Drug Relief Foundation, 10F., No. 22, Aiguo E. Rd., Zhongzheng Dist., Taipei City, 100, Taiwan,* *wwchen@tdrf.org.tw; ^d^Department of Medical Affairs, Ministry of Health and Welfare, No. 488, Section 6, Zhongxiao E Rd, Nangang District, Taipei City, 115, Taiwan, dtemer14@gmail.com; ^e^Ministry of Health and Welfare, No. 488, Section 6, Zhongxiao E Rd, Nangang District, Taipei City, 115, Taiwan,* *md01@mohw.gov.tw; ^f^Department of Family and Community Medicine, Tri-Service General Hospital and School of Medicine, National Defense Medical Center,* *No.325, Sec. 2, Chenggong Rd., Neihu Dist., Taipei City, 114, Taiwan, College of Law, National Chengchi University, No.64, Sec.2, ZhiNan Rd., Wenshan District, Taipei City, 116, Taiwan, darthravenhugo@gmail.com; ^g^Department of Family and Community Medicine, Tri-Service General Hospital and School of Medicine, National Defense Medical Center, No.325, Sec. 2, Chenggong Rd., Neihu Dist., Taipei City, 114, Taiwan, tsghccwang@gmail.com*

***Corresponding author:** Chih-Chia Wang

Department of Family and Community Medicine, Tri-Service General Hospital and School of Medicine, National Defense Medical Center
No.325, Sec. 2, Chenggong Rd., Neihu Dist., Taipei City, 114, Taiwan
Phone No: +886-2-8792-3311
Fax No: +886-2-8792-7207
Email Address: tsghccwang@gmail.com

Contents

Supplementary 1. Mediation process for medical disputes in Taiwan.………………………………………………………1

Supplementary 2. Parts of the medical dispute training component in the workshop…………………………………………2

**Supplementary 1. Mediation process for medical disputes in Taiwan**

**
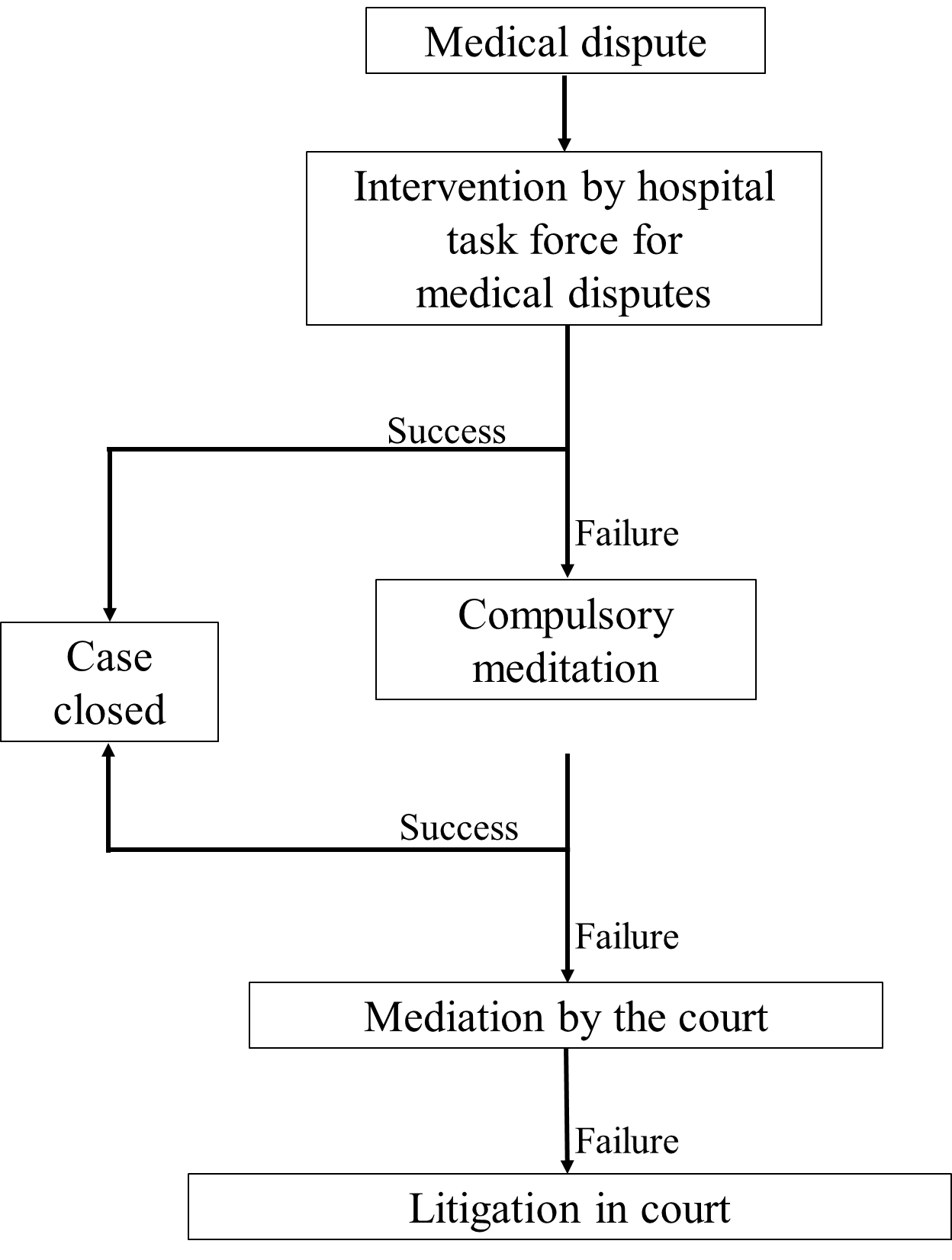
**

**Supplementary 2. Parts of the medical dispute training component in the workshop**

| Part 1: Lectures (1.5 hour)   - Consists of four parts:  1. Medical disputes mediation mechanism: an overview of the status of medical dispute management was provided. The participants analyzed the pros and cons of litigation and understood the similarities and disparities in ADR. 2. Procedures of medical dispute mediation: trainees comprehended the proceeding of medical dispute mediation and learned the core elements of mediation statements. 3. Dual-mediator system: trainees learned the operation mechanism and tips for cooperation between mediators. They also learned how to identify potential problems and to seek for government support. 4. Mediation principles and techniques: the participants were familiarized with the standards of issues regarding medical dispute mediation and mediation skills, such as listening, reshaping, and body language. They were encouraged to implement these skills in practice.   Part 2: Group discussions (1 hour)   - How to put the mediation skills into practice and report the process of medication officially   Part 3: Case practice (1.5 hour)  A situation-based scenario, which was adapted from an actual medical dispute, was presented to the trainees to simulate the mediation process. The trainees role-played various parties and acted as mediators to harmonize disputes.   - A surgical medical dispute draft was provided to six trainees who were assigned to a group. The trainees played the roles of mediation committee members, doctors, and patients, and the instructors gave directions on mediation skills. After the role play, members shared their opinions during group discussions. |
| --- |
